# Antioxidants Trolox and Methazolamide Protect Microvascular Endothelial Cells from Oxidative Damage Induced by Sporadic and Familial Forms of Oligomeric Amyloid-β

**DOI:** 10.3390/antiox14111375

**Published:** 2025-11-19

**Authors:** Maria Luisa Valle, Bitseat Getaneh, Christopher William, Jorge Ghiso, Agueda Rostagno

**Affiliations:** Department of Pathology, New York University Grossman School of Medicine, New York, NY 10016, USA; maria.valle@nyulangone.org (M.L.V.); bkg18@georgetown.edu (B.G.); christopher.william@nyulangone.org (C.W.)

**Keywords:** Alzheimer’s disease, cerebral amyloid angiopathy, oxidative stress, ROS, Aβ oligomers

## Abstract

Cerebral amyloid angiopathy (CAA), present in more than 90% of Alzheimer’s disease (AD) cases, associates with focal ischemia and neurovascular dysfunction. Genetic variants at positions 21–23 of amyloid beta (Aβ), among them the Dutch mutation (AβE22Q), are primarily linked to CAA and the development of cerebral hemorrhages. An important contributor to CAA pathogenesis is the dysregulation of mitochondria-mediated pathways with concomitant induction of oxidative stress. Using biochemical assays and immunofluorescence microscopy, this work demonstrates the exacerbated formation of reactive oxygen species (ROS) in human brain microvascular endothelial cells after short exposure to soluble oligomers of synthetic homologues of Aβ1-42 and the Dutch variant, inducing lipid peroxidation and protein carbonylation, both markers of oxidative stress. The heterogeneity of the soluble oligomeric assemblies inducing this oxidative response was highlighted by their reactivity with two conformational antibodies recognizing specific and mutually exclusive epitopes associated with either soluble prefibrillar oligomers or soluble fibrillar oligomers. Treatment with the multitarget antioxidants Trolox and methazolamide significantly attenuated the Aβ-mediated ROS production and reduced oxidative stress markers to basal levels. Our data highlight the damaging role of heterogeneous Aβ oligomers and the preventing effect of antioxidants, suggesting ROS modulation as a complementary therapeutic strategy to preserve neurovascular unit integrity.

## 1. Introduction

**Figure 1:**
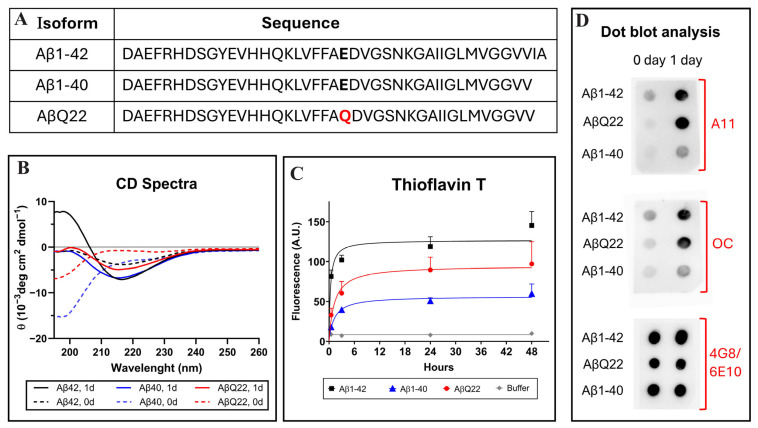
Structural and aggregation properties of Aβ1-42, Aβ1-40, and the AβQ22 Dutch variant. (**A**) Amino acid sequence of the different Aβ peptides. Residues at position 22 are indicated in bold type and the Dutch amino acid substitution is highlighted in red. (**B**) Changes in the secondary structure of Aβ1-40 (blue lines), Aβ1-42 (black lines), and AβQ22 (red lines) peptides, assessed by circular dichroism spectrometry in the far-UV spectra (195–260 nm). Broken lines illustrate spectra immediately after peptide solubilization (zero time) and continuous lines depict changes after 24 h of incubation in PBS. Data represent the average of 15 consecutive spectra and the results are expressed in molar ellipticity. (**C**) Aggregation of Aβ homologues monitored by fluorescence evaluation of Thioflavin T binding up to 48 h of incubation in PBS. Data represent the mean ± SEM of at least three independent experiments. (**D**) Dot blot identifying the presence of soluble oligomeric species in the different Aβ preparations evaluated through immunoreactivity with polyclonal anti-prefibrillar oligomers A11 (top panel) and polyclonal anti-fibrillar oligomers OC (middle panel). Reactivity with a mixture of anti-Aβ monoclonals 4G8 and 6E10 was used as a loading control (bottom panel).

Alzheimer’s disease (AD) is recognized as the most common form of dementia among the elderly population over 65 years of age. The disorder is neuropathologically characterized by synaptic loss, the presence of hyperphosphorylated intraneuronal neurofibrillary tangles, and the accumulation of amyloid beta (Aβ) deposits within the brain parenchyma and cerebral vessels. Cerebrovascular deposition of amyloid, known as cerebral amyloid angiopathy (CAA), present in >90% of AD cases, is the most frequent condition associated with focal ischemia, cerebral hemorrhage, and neurovascular dysfunction [1]. While the main Aβ isoform contributing to the vascular deposits is Aβ1-40, these lesions also contain the longer peptide Aβ1-42—the proteoform most abundant in neuritic plaques—although the ratio Aβ1-40:Aβ1-42 is higher in the vascular deposits than in parenchymal lesions [2,3,4,5]. Notably, Aβ1-42 is particularly abundant in amyloid deposits, compromising brain capillaries, which severely affect BBB function, increasing barrier permeability, and contributing to cognitive impairment and the development of cerebral hemorrhages [6]. The extent of amyloid vascular compromise is exacerbated by the presence of Aβ genetic variants, particularly those located at positions 21–23 of the molecule, which have been consistently associated with CAA, hemorrhagic stroke, and dementia [1,7]. The first described genetic variant was reported in 1990 in members of a Dutch kindred exhibiting an early onset and a very aggressive form of CAA known as Hereditary Cerebral Hemorrhage with Amyloidosis–Dutch type (HCHWA-D) [8]. The Dutch mutation (AβE22Q) results from a single nucleotide transversion (G for C) at codon 693 of the precursor protein APP, leading to the E > Q amino acid substitution at position 22 of the Aβ molecule (Figure 1A) [8]. The loss of a negatively charged residue resulting from this substitution translates in a dramatic acceleration of Aβ aggregation with impaired transport and brain clearance mechanisms that lead to increased cerebrovascular amyloid accumulation through mechanisms still not fully understood [7,9].

Amyloid deposition in the vessel wall of leptomeningeal arteries and arterioles takes place gradually. Aβ deposition initially accumulates in the basement membrane surrounding smooth muscle cells—decreasing cell viability and vascular reactivity—and progressively spreads toward the endothelium [4,10,11,12]. Despite being less affected by the initial Aβ deposition in larger vessels, cerebral endothelial cells (ECs)—the focus of the current study—are the main cellular elements compromised in capillary CAA and crucial contributors to disease pathogenesis actively participating in brain clearance mechanisms and BBB function, both known to be impaired in AD patients [4,13]. Indeed, ECs play a major role in the brain clearance of Aβ to the blood, a mechanism mediated by efflux transporters abundant in brain capillary endothelium [14,15]. As the interface between blood and brain, the cerebral endothelium is the major component of the BBB, a highly selective barrier strengthened by the formation of tight junctions between ECs. Vascular accumulation of Aβ severely compromises BBB function, allowing influx of inflammatory cells, exacerbating neuroinflammation, and increasing the occurrence of microbleeds [7,13,16,17]. Ours and others’ experimental work has demonstrated the ability of aggregated forms of Aβ peptides, particularly Aβ1-42 and AβE22Q, to increase endothelial barrier permeability in vitro, an effect correlated with changes in the expression of the occludin, claudin-5, and ZO-1 tight junction proteins [10,18,19,20].

In the context of the current work, it is noteworthy that oxidative stress has been shown as a key contributor to microvascular EC damage, BBB permeability changes, and the development of microhemorrhages. EC apoptotic changes, as well as EC release of pro-inflammatory cytokines and extracellular matrix proteases capable of tight junction protein degradation, are some of the ROS-associated elements described as contributing factors for microbleed and microhemorrhage formation [21,22,23]. Based on the relevance of oxidative stress for AD pathogenesis and the detrimental role of ROS in cell culture and animal models, several studies have focused their attention on developing pharmacological agents targeting these mechanisms as a therapeutic strategy. Among the multiple compounds tested, numerous phytochemicals and natural antioxidants have proved successful in various neuronal and vascular cell culture models [24,25,26,27,28,29]. Different chemical modifications of these compounds have also been studied, including the use of synthetic homologues with increased water solubility for improved bioavailability and cellular accessibility. In this context, Trolox (TX), a synthetic hydrophilic water- and lipid-soluble analog of the lipophilic α-tocopherol, was shown to exhibit superior radical scavenging efficacy and improved antioxidant properties compared to its unmodified vitamin E counterpart [30], attenuating Aβ-mediated toxicity in neuronal and vascular cells [30,31,32,33,34]. Numerous other compounds have also been evaluated for their ability to counteract the detrimental effect of Aβ in different models, among them inhibitors of carbonic anhydrases. Our group and others have shown the anti-apoptotic properties of the carbonic anhydrase inhibitor methazolamide (MTZ) on neuronal, glial, and endothelial cells challenged with Aβ1-42 and the AβE22Q Dutch variant [35,36]. These studies demonstrated that MTZ supplementation prevented cytochrome c release to the cytoplasm, inhibited caspase activation, and protected cerebral cells from the induction of Aβ-mediated apoptotic mechanisms [35,36,37]. Early studies indicated that MTZ also exhibited antioxidant properties, protecting neuronal cells from H_2_O_2_-induced oxidative damage and increasing cell survival [38]. More recent research has shown that the compound prevented mitochondrial membrane depolarization and inhibited the production of mitochondrial H_2_O_2_ following Aβ treatment in neuronal cells and ECs [35,39]. Recently, we reported that the protective effect of TX and MTZ from Aβ-mediated ROS generation in SH-SY5Y cells and rat primary cortical neurons involved activation of the transcription factor nuclear factor erythroid 2-related factor 2 (Nrf2)—a master regulator of antioxidant cellular responses [40,41]—through the phosphoinositide 3-kinase (PI3K)/Akt axis [42].

Despite mounting evidence demonstrating an important role for the Aβ-mediated generation of oxidative stress in the alterations of the cerebral endothelium function, as indicated above, limited information is available regarding the changes induced by AβE22Q, not whithstanding the numerous studies involving this variant in EC apoptosis [9,21,35,43]. Furthermore, while the effect of ROS generation on lipid peroxidation and protein carbonylation has been extensively studied in the context of AD, this previous work mostly addresses these molecular modifications in the context of neuronal dysfunction and cognitive impairment and they have not been shown in ECs [44,45]. The present work expands the knowledge on the effect of sporadic forms of Aβ, as well as of the familial AβE22Q variant, on ROS formation in microvascular ECs and concomitant downstream oxidative molecular changes, providing insight into the structural composition of the oligomeric assemblies inducing these modifications. The observed quenching of the oxidative changes exerted by the antioxidants TX and MTZ suggests a potential for these pharmacological compounds as complementary therapeutic agents for the preservation of function and integrity of the neurovascular unit.

## 2. Materials and Methods

### 2.1. Materials

Methazolamide (MTZ; N-[5-(aminosulfonyl)-3-methyl-1,3,4-thiadiazol-2(3H)-ylidene]-acetamide) and Trolox (TX; 6-hydroxy-2,5,7,8-tetramethylchroman-2-carboxylic acid) were procured from Millipore-Sigma (Burlington, MA, USA).

### 2.2. Synthetic Peptides

Synthetic homologs of Aβ1-42, Aβ1-40, and the Aβ1-40E22Q (from here on referred to as AβQ22) variant were synthesized using N-tert-butyloxycarbonyl chemistry at ERI Amyloid Laboratory (Oxford, CT) and purified by reverse-phase high-performance liquid chromatography, as previously reported [43]. Peptide masses were corroborated by matrix-assisted laser desorption ionization time-of-flight (MALDI-TOF) mass spectrometry, and concentrations were evaluated by amino acid analysis, as previously described [46,47]. Peptides were dissolved at a concentration of 1 mg/mL in 1,1,1,3,3,3, hexafluoro-isopropanol (HFIP; Sigma Chemical Co., St. Louis, MO, USA) and incubated overnight at room temperature. Following lyophilization, peptides were thoroughly dissolved to 5 mM in dimethyl sulfoxide (DMSO; Sigma), brought up to 1 mM with PBS, and further diluted in basal culture media to the desired concentration for all the cell culture experiments.

### 2.3. Thioflavin T Binding Assay

Thioflavin T binding was monitored as described previously [43]. For each aggregation time point (0.5, 3, 24, and 48 h), 6 μL aliquots from each peptide (1 mg/mL) were mixed with 184 μL of 50 mM Tris–HCl buffer pH 8.5 and 10 µL of freshly prepared 0.1 mM Thioflavin T (Sigma). As blanks for each aggregation point, the 6 µL sample volume was replaced by buffer. In all cases, fluorescence was recorded after 300 s in a LS-50B luminescence spectrometer (Perkin Elmer, Waltham, MA, USA) with excitation and emission wavelengths of 435 and 490 nm, respectively, and a slit width of 10 nm. Each sample was analyzed in duplicate.

### 2.4. Circular Dichroism Spectroscopy

Changes in the secondary structure of Aβ1-42, Aβ1-40, and the Dutch variant AβQ22 were estimated by circular dichroism (CD) spectroscopy, as previously described [46,47,48]. All peptides were pretreated overnight with HFIP, as described above, followed by lyophilization and further solubilization in 10 mM phosphate buffer (pH 7.4) containing 150 mM sodium fluoride to a final concentration of 50 μM. Spectra in the far-ultraviolet (wavelength range: 195–260 nm; bandwidth: 1 nm; intervals: 1 nm; scan rate: 60 nm/min) were recorded for each peptide immediately after solubilization and following 24 h of incubation at 37 °C in a Jasco J-720 spectropolarimeter (Jasco Inc., Easton, MD, USA) using a 1 mm-path quartz cell. Fifteen consecutive spectra from each sample were averaged, subtracted from baseline values, and results were expressed in molar ellipticity terms (deg cm^2^ dmol^−1^)

### 2.5. Assessment of Peptide Conformations by Dot Blot Analysis

The presence of prefibrillar and fibrillar oligomeric forms in the different synthetic homologue preparations was assessed by evaluating their reactivity in dot blot analysis using rabbit polyclonal OC anti-fibril antibody (Millipore Sigma)—recognizing fibrillar oligomers and mature fibrils but not monomers or prefibrillar oligomers [49]—and rabbit polyclonal A11 anti-oligomer antibody (ThermoFisher Scientific/Invitrogen, Waltham, MA, USA)—recognizing prefibrillar oligomers but not mature fibrils or monomers [50]—following previously described protocols [40,47,48]. Briefly, 100 µL of each 50 µM peptide preparation—representing 22.57 µg for Aβ1-42 and 21.65 µg for Aβ1-40 and AβQ22—both freshly solubilized and after 24 h of incubation in PBS, mimicking the cell culture experimental conditions, as indicated above, were loaded on a 0.45 μm nitrocellulose membrane (Biorad, Hercules, CA, USA) assembled into a Bio-Dot Microfiltration Apparatus (Bio-Rad). Samples were allowed to diffuse passively for 1 h before vacuum application and blocking in situ for 1 h with 1% nonfat milk in tris-buffered saline (TBS) containing 0.1% Tween (TBST). After removing from the dot blot unit, the membranes were subsequently washed with TBST, blocked for an additional hour with 5% milk in TBST, and incubated overnight at 4 °C with OC antibodies (1:1000 dilution). After washing with TBST, the membrane was further reacted overnight at 4 °C with HRP-conjugated goat anti-rabbit secondary antibody (ThermoFisher Scientific; 1:10,000). All incubation and washing steps were conducted under gentle shaking. Immunoreactivity was evaluated by enhanced chemiluminescence (ECL; ThermoFisher Scientific) using the ChemiDoc MP imaging system (Bio-Rad). To detect the presence of prefibrillar oligomers in the different peptide preparations, the membrane was stripped for 15 min with Restore Western Blot Stripping Buffer (ThermoFisher Scientific) and further incubated with A11 antibody (1:1000 dilution), followed by HRP-conjugated anti-rabbit antibody and ECL evaluation, as above. As a control, and to illustrate peptide load, the membrane was stripped in the same conditions as above and further incubated with a 50:50 combination of mouse monoclonal 4G8 and 6E10 anti-Aβ antibodies (BioLegend San Diego, CA, USA; 1:3000 each), recognizing residues 17–24 and 1–16, respectively. Immunoreactivity with these pan-Aβ antibodies was assessed by 1 h of incubation with HRP-conjugated goat anti-mouse antibody (Invitrogen, 1:30,000), followed by ECL and signal evaluation, as above.

### 2.6. Cell Culture Experiments

Human brain microvascular endothelial cells (hCMEC/D3, referred herein as ECs) were obtained from Babette Weksler (Cornell University, Ithaca, NY, USA). The cell line has been extensively characterized and is a well-established model of human BBB function [51]. Cells were grown in EBM2 media (Lonza, Walkersville, MD, USA) supplemented with 2.5% FBS and SingleQuots Supplements (fibroblast growth factor, epidermal growth factor, vascular endothelial growth factor, insulin-like growth factor, hydrocortisone, ascorbic acid, and gentamycin) specifically formulated for optimal growth and maintenance of microvascular ECs. Cells were switched to basal EBM2 media containing 1% FBS, as described [43], and after 1 day of rest, challenged for 24 h with either wildtype Aβ1-42, Aβ1-40, or AβQ22 peptides at a final concentration of 50 μM. Incubation time with the synthetic homologues was not extended for more than 24 h, as cell death was observed with more prolonged incubations (Supplementary Figure S1), in agreement with previous reports by our group [43,46,48,52].

In the studies assessing the ameliorating effect of antioxidant compounds, cells were pretreated with either 300 μM TX or 300 μM MTZ for 2 h before the addition of the respective Aβ peptides, as described [42]. Whole cell lysates (WCLs) were prepared using ice-cold modified RIPA buffer (50 mM TRIS Base, 150 mM NaCl, 1 mM EDTA, 1% NP40, 0.5% sodium deoxycholate, 0.1% SDS, pH 7.5), as previously reported [53]. Cell lysates were centrifuged at 10,000 rpm for 15 min and stored at −20 °C until analysis. Protein concentration was assessed using the bicinchoninic acid (BCA) protein assay (ThermoFisher Scientific).

### 2.7. Detection of Reactive Oxygen Species

ROS formation was evaluated by immunofluorescence (IF) using CellROX Deep Red dye (ThermoFisher Scientific), a probe recognizing different ROS species, including hydrogen peroxide, as well as hydroxyl and superoxide radicals, following previously described protocols [42]. ECs were seeded on collagen-coated 96-well plates, rested for 24 h, and further incubated for 24 h with the different synthetic homologues in the presence or absence of antioxidant compounds. Following treatment with the different peptides, cells were subsequently incubated with CellROX Deep Red dye (5 μM) and the nuclear counterstain dye Hoechst (0.25 μg/mL) for 30 min at 37 °C, followed by a 15 min fixation in 4% paraformaldehyde. Images were captured in a Nikon Eclipse Ti microscope (Nikon Instruments Inc., Melville, NY, USA) and analyzed using ImageJ (Version 1.53t) (Fiji plugin version 2.14, https://fiji.sc/, accessed on 13 November 2025).

The generation of reactive oxygen and nitrogen species induced by the different Aβ peptides, including hydrogen peroxide, nitric oxide, peroxyl radical, and peroxynitrite anions, was assessed by ELISA using the DCF ROS/RNS assay (Abcam), as previously described [54]. The test, which employs the fluorogenic probe dichlorodihydrofluorescin DiOxyQ (DCFH-DiOxyQ), was performed in WCLs from ECs challenged with the different Aβ peptides in the presence or absence of the antioxidants TX and MTZ following the manufacturer’s protocol. Briefly, 50 µL of each EC-derived WCL (1:100 dilution) and standards were combined with 50 µL of catalyst and incubated for 5 min at room temperature. This was followed by the addition of 100 µL of the fluorogenic probe and 30 min room-temperature incubation. Fluorescence, which is proportional to the total ROS/RNS levels within the sample, was evaluated 480 nm/530 nm (excitation/emission) in a Synergy™ Neo2 Multi-Mode Microplate Reader (Agilent, Santa Clara, CA, USA) and data were normalized to mg of protein content.

### 2.8. Detection of Lipid Peroxidation

ROS-induced lipid peroxidation was assessed by IF using the lipid peroxidation assay kit (Abcam, Cambridge, MA, USA), which uses a sensitive radiometric lipid peroxidation sensor. This sensor changes its fluorescence from red to green upon ROS-mediated peroxidation, allowing—through this shift—the radiometric evaluation measurement of lipid peroxidation. ECs were seeded on collagen-coated 96-well plates and incubated for 24 h with the different Aβ synthetic homologues, in the presence or absence of the antioxidant compounds, as described above. Following Aβ challenge, cells were incubated with 1X lipid peroxidation sensor and the nuclear dye Hoechst (0.25 μg/mL) for 30 min at 37 °C. Images were captured using a Nikon Eclipse Ti microscope and analyzed using ImageJ.

Lipid peroxidation was additionally assessed by ELISA, assessing the formation of malondialdehyde (MDA) by ELISA (MDA Assay Kit, Abcam). The test evaluates the levels of MDA, a reactive aldehyde resulting from the peroxidation of polyunsaturated fatty acids and a commonly used biomarker of oxidative stress. MDA-protein adducts were quantitated in WCLs from ECs incubated with the different Aβ peptides, as described above, following the manufacturer’s protocol.

### 2.9. Detection of Protein Carbonylation

The presence of protein oxidation derivatives was determined, assessing the formation of DNP hydrazones generated by the reaction of 2,4-dinitrophenylhydrazine (DNPH) with protein carbonyls by both dot blot (Abcam) and ELISA (Abcam). Endothelial cells were seeded on collagen-coated 6-well plates, and after 24 h of adaptation, challenged with the different Aβ synthetic homologues in the presence or absence of the antioxidant compounds for an additional 24 h, as described above. Following treatment with the Aβ peptides, ECs were lysed and incubated with DNPH, in accordance with the pertinent assay instructions.

For dot blot analysis, cells were lysed with the extraction buffer (1X) provided with the kit and protein concentration assessed using the BCA protein assay. Samples, at a concentration of 500 µg/mL, were derivatized with DNPH solution according to the manufacturer’s instructions and loaded onto a 0.45 µm nitrocellulose membrane assembled into a dot blot apparatus, as described above, for the detection of oligomeric species. Following sample application, membrane blocking, and TBST washes, the membrane was incubated overnight at 4 °C with the anti-DNPH primary antibody provided in the kit. The following day, the membrane was washed with TBST and incubated for 1 h at room temperature with the HRP-conjugated secondary antibody included in the kit. Immunoreactivity was evaluated by ECL, as above, and the intensity of each dot was evaluated using ImageJ Fiji and normalized to mg of protein.

For ELISA assessment, WCLs at a 500 µg/mL concentration were derivatized with DNPH solution following the manufacturer’s instructions. The formation of DNP hydrazone adducts, proportional to the carbonyls present in each sample, was detected spectrophotometrically at 375 nm using a Synergy™ Neo2 Multi-Mode Microplate Reader. Carbonyl content (nmol/well) was normalized to mg of protein.

### 2.10. Statistical Analysis

Statistical analysis was performed with GraphPad Prism 9 using one-way ANOVA with Tukey’s multiple comparison tests. Outliers were identified and excluded from the dataset via the ROUT method (Q = 10%). Values of *p* < 0.05 were considered significant. Data represent the mean ± standard deviation of at least 3 independent replicates.

## 3. Results

### 3.1. Structural and Aggregation Properties of Aβ1-42, Aβ1-40, and AβQ22 Dutch Variant

Circular dichroism (CD) spectroscopy, Thioflavin T binding assay, and assessment of oligomeric species by dot blot were performed to evaluate structural characteristics and oligomerization/fibrillization properties of the different Aβ homologues used in the study (Figure 1). CD scans illustrate the secondary structure of Aβ1-42, Aβ1-40, or AβQ22 at zero time (broken lines) and after 24 h of incubation in PBS (solid lines). Immediately after solubilization, Aβ1-40 and AβQ22 adopted a typical unordered conformation with a classic CD minimum at 198 nm, characteristic of random conformations, while Aβ1-42 showed a mixture of random structures with still negative values at 198 nm and β-sheet-rich conformers, exhibiting a typical minimum at 218 nm. After 24 h of incubation, both Aβ1-40 and AβQ22 peptides, although still maintaining random conformers, showed formation of β-sheet structures, whereas—at this time point—β-sheet conformations were the predominant components in Aβ1-42 (Figure 1B). Comparative oligomerization/fibrillization characteristics of the three Aβ homologues reconstituted in PBS were monitored through changes in Thioflavin T fluorescence, as shown in Figure 1C. The binding of Thioflavin T to newly formed β-sheet structures, monitored for up to 48 h, showed differential enhanced fluorescence of Aβ1-42 > AβQ22 > Aβ1-40, with Aβ1-42 reaching a plateau in about 6 h, and the AβQ22 and Aβ1-40 homologues requiring additional time. The presence of soluble prefibrillar and fibrillar oligomeric forms of the peptides was evaluated by assessing their reactivity with A11 anti-oligomer and OC anti-fibril antibodies, respectively, by dot blot. As illustrated in Figure 1D (top panel), polyclonal A11 showed strong immunoreactivity at 24 h for both Aβ1-42 and AβQ22, whereas prefibrillar oligomeric forms of Aβ1-40 were virtually absent in the same experimental timeframe. A similar pattern of immunoreactivity was obtained using polyclonal OC as the primary antibody, indicating the presence of soluble fibrillar oligomers in the Aβ1-42 and AβQ22 preparations and the almost negligible reactivity in the Aβ1-40 sample (Figure 1D, middle panel). As a control for peptide load, the membrane was probed with a mixture of anti-Aβ monoclonals 4G8 and 6E10, recognizing all the peptide preparations (Figure 1D, bottom panel). Overall, and consistent with our previous reports, Aβ1-42 and AβQ22 showed enhanced oligomerization/fibrillization properties compared to Aβ1-40, forming both prefibrillar and fibrillar types of oligomeric assemblies within the 24 h experimental timeframe, paralleling the magnitude of newly formed β-sheet structures observed by Thioflavin T and CD spectroscopy (Figure 1B,C) [43,47,48,49,50].

### 3.2. Aβ-Mediated ROS Generation in Cerebral Microvascular ECs and Protection by Antioxidants

**Figure 2:**
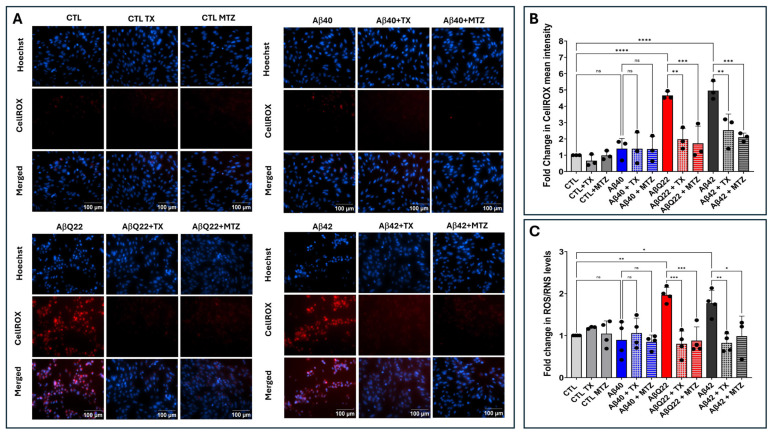
Aβ-mediated ROS generation and protection by antioxidants. Endothelial cells were challenged for 24 h with Aβ1-42, Aβ1-40, and AβQ22 synthetic homologues at a 50 μM final concentration, in the presence or absence of the antioxidants MTZ and TX. (**A**) Cells stained with CellROX Deep Red with Hoechst nuclear counterstaining. Bars indicate 100 µm for all images. (**B**) Quantification of CellROX fluorescence assessed via ImageJ Fiji software (Version 1.53t). (**C**) Changes in ROS/RNS levels evaluated by ELISA. In (**B**,**C**) data are expressed as fold change compared to untreated control cells. In both cases, graphs depict mean plus minus SD and black dots illustrate individual data points. * Indicates *p* < 0.05, ** *p* < 0.01, *** *p* < 0.001, and **** *p* < 0.0001, ns: *p* > 0.05.

Oxidative stress and mitochondria-generated ROS have been associated with the progression of several neurodegenerative disorders [55,56]. Cerebral microvascular endothelial cells were challenged for 24 h with synthetic homologues of Aβ1-40, Aβ1-42, and AβQ22 at 50 µM, a concentration frequently used in in vitro assays measuring induction of cell death and oxidative stress mechanisms as they generate strong reproducible toxic responses [35,43,48,57,58]. IF microscopy studies indicated that ECs exposed to Aβ1-42 and the AβQ22 variant—in contrast to those challenged with Aβ1-40—displayed abundant ROS formation, as highlighted by the free radical sensing fluorogenic probe CellROX (Figure 2A,B). It should be noted that the increased ROS formation by AβQ22 assessed by CellROX fluorescence microscopy in the current work contrasts with previously published research in ECs challenged with comparable Aβ homologues [35]. Whether this reflects different experimental conditions or the use of different readout platforms remains to be determined. In this sense, it is noteworthy to mention that, although with lower throughput than reader-based methodologies, microscopy-based assessment may not only exhibit higher sensitivity but also prove beneficial in the evaluation of non-homogeneous cellular samples [59,60,61]. Treatment with the antioxidants MTZ and TX for 2 h prior to the addition of the Aβ1-42 and AβQ22 homologues significantly attenuated ROS formation, bringing CellROX fluorescence signals to the levels of control EC that had not sustained challenge with the Aβ peptides (Figure 2A,B).

The production of free radicals induced by the different Aβ peptides was also evaluated by ELISA using the DCF ROS/RNS assay. Confirming the IF microscopy data, this test demonstrated a ~2-fold increase in ROS/RNS levels in AβQ22-treated ECs and a ~1.8-fold increase in cells challenged with Aβ1-42, compared to untreated controls (Figure 2C). In contrast, incubation with Aβ1-40 did not induce significant changes in ROS/RNS levels. Treatment with MTZ and TX prior to the addition of the Aβ1-42 and AβQ22 homologues significantly attenuated ROS/RNS formation, bringing the fluorescence intensity to levels comparable to those of control cells not incubated with the Aβ peptides (Figure 2C).

It should be noted that the 50 µM peptide preparations used in the current work for the assessment of ROS formation by both IF and ELISA contained soluble prefibrillar and fibrillar oligomers, as demonstrated by the dot blot assays, among other undetermined Aβ soluble structures. As a result, the concentration of each type of assembly, although undefined, is likely to be lower than the total 50 µM levels in the preparation. The use of purified prefibrillar and fibrillar oligomers in a wider concentration span—extending into lower µmolar ranges than the ones employed herein—will more precisely define the oligomeric structural and concentration requisites for ROS generation.

Interestingly, the ROS formation results correlate with the aggregation/oligomerization propensity of the different Aβ peptides illustrated in Figure 1. The lack of ROS formation induced by Aβ1-40 after 24 h challenge is consistent with our previously published work on EC apoptosis, in which we demonstrated that, despite leading to the endpoint activation of analogous cell death pathways, the AβQ22 variant showed an accelerated and enhanced response correlating with the early onset of the disease. In contrast, Aβ1-40 required longer experimental timeframes, matching up the time requirement for the formation of aggregated, oligomeric/prefibrillar structures and the association with the late-onset clinical phenotype of sporadic CAA [43,48]. Further studies using pre-formed Aβ1-40 aggregated/oligomeric assemblies to challenge EC cultures are required to determine whether this is also the case for the induction of oxidative stress mechanisms.

### 3.3. Aβ-Mediated Lipid Peroxidation in Cerebral Microvascular ECs and Protection by Antioxidants

**Figure 3:**
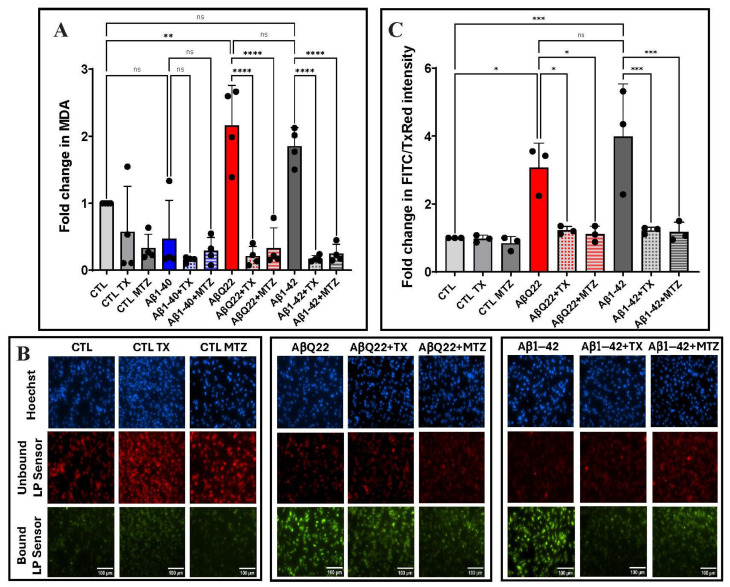
Aβ-mediated ROS lipid peroxidation and protection by antioxidants. Endothelial cells were treated for 24 h with Aβ1-42, Aβ1-40, and AβQ22 peptides at a 50 μM final concentration, in the presence or absence of the antioxidants MTZ and TX. (**A**) Lipid peroxidation in EC lysates assessed through evaluation of MDA levels. (**B**) Representative IF images of lipid peroxidation evaluated after staining with the lipid peroxidation sensor, which shifts its fluorescence from red to green upon peroxidation. Bars represent 100 µm in all cases. (**C**) Quantification of fluorescence intensity of the lipid peroxidation sensor was normalized to the unbound dye using ImageJ Fiji software. In A and C data are expressed as fold change compared to untreated control cells. In both cases, graphs depict mean ± SD and black dots illustrate individual data points. * Indicates *p* < 0.05, ** *p* < 0.01, *** *p* < 0.001, and **** *p* < 0.0001, ns: *p* > 0.05.

The extent of lipid peroxidation in cells and tissues is an additional indicator of oxidative stress and cellular damage [62]. Lipid peroxidation mainly results from the reaction of free radicals with polyunsaturated fatty acids in cell membranes and leads to the formation of different lipid peroxidation products, including lipid hydroperoxides and MDA. EC lysates, treated separately with 50 μM Aβ1-42, Aβ1-40, or AβQ22, in the presence or absence of MTZ and TX, were analyzed by ELISA to assess MDA levels. A ~2-fold increase was observed in EC challenged with Aβ1-42 and the AβQ22 variant compared to non-Aβ-treated control cells (Figure 3A). No significant change in MDA was observed in Aβ1-40-treated cells, consistent with the absence of ROS generation illustrated in Figure 2. Co-incubation with both MTZ and TX counterbalanced the increase in MDA levels (Figure 3A).

Lipid peroxidation was additionally evaluated by immunofluorescence in hCMEC/D3 cells challenged with Aβ1-42 and AβQ22 variant peptides, cells that displayed elevated MDA levels, as indicated above. Exposure to either peptide resulted in a 3- and 4-fold increase in lipid peroxidation levels observed as a green fluorescence signal, in contrast to untreated controls in which the lipid peroxidation sensor is mainly visualized in its unbound form as a red fluorescence signal. Co-incubation with antioxidants TX and MTZ significantly rescued the ROS-induced lipid peroxidation at comparable levels (Figure 3B,C).

### 3.4. Aβ-Mediated Protein Carbonylation in Cerebral EC and Protection by Antioxidants

**Figure 4:**
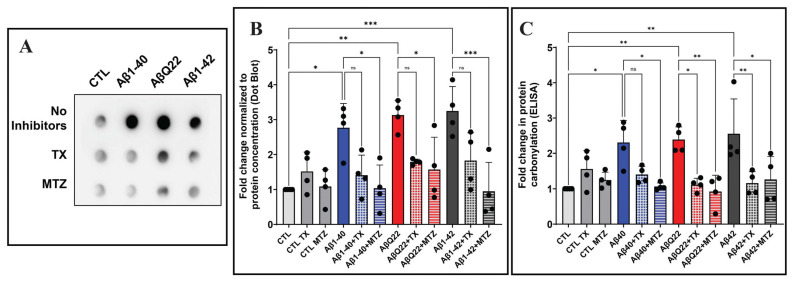
Aβ-mediated carbonylation and prevention by antioxidants. Endothelial cells were treated with 50 μM Aβ1-42, Aβ1-40, or AβQ22 in the presence or absence of 300 μM MTZ or 300 μM Trolox for 24 h. The protein carbonyl content, proportional to the formation of DNP hydrazones upon their reaction with 2,4-dinitrophenylhydrazine (DNPH), was estimated in cell lysates via dot blot and ELISA. (**A**) Representative dot blot showing changes in DNP levels in cells challenged with Aβ1-42, Aβ1-40, or AβQ22 in the presence or absence of TX or MTZ. (**B**) The signal intensity of each dot was quantitated using ImageJ Fiji and normalized to the protein concentration. (**C**) Assessment of protein carbonylation by ELISA normalized by protein concentration and expressed as nmol carbonyl/mg protein. In (**B**,**C**), data are expressed as fold change compared to untreated controls. In both cases, graphs depict mean ± SD and black dots illustrate individual data points. * Indicates *p* < 0.05, ** *p* < 0.01, and *** *p* < 0.001, ns: *p* > 0.05.

Protein carbonylation refers to a type of protein modification that can be promoted by reactive oxygen or nitrogen species [63]. Following EC incubation with Aβ1-42, Aβ1-40, or the AβQ22 variant—in the presence or absence of the antioxidants MTZ or TX—protein carbonyl content was estimated in the respective cell lysates via dot blot and ELISA. Both methodologies are based on the reaction of 2,4-dinitrophenylhydrazine (DNPH) with protein reactive aldehyde or ketone residues to form stable DNP hydrazone adducts in a proportional rate to the carbonyls present [63]. Dot blot analysis shows that all Aβ species tested induced a ~3-fold increase in protein carbonylation, compared to non-peptide controls. In contrast to the lipid peroxidation experiments, ECs challenged with Aβ1-40 induced significant protein carbonylation levels even when ROS formation was not detected. These results may reflect the higher sensitivity of the methodologies used to assess DNP content and/or they may relate to a lower degradation rate of carbonylated proteins, likely leading to their cellular accumulation and their increased detection rate [64,65]. In all cases, the increased DNP hydrazones content generated by the synthetic homologues was significantly attenuated upon the addition of the antioxidants MTZ or TX (Figure 4A,B). The increase in protein carbonylation was further confirmed via ELISA, which demonstrated a ~2-fold increase in protein carbonylation after challenge with all Aβ peptides tested. As above, supplementation with the antioxidants MTZ and TX significantly decreased DNP hydrazones formation in all cases (Figure 4C).

## 4. Discussion

CAA, a well-recognized cause of intracerebral hemorrhage, is also a major contributor to ischemic stroke and dementia [3]. Among the many molecules known to be associated with the formation of cerebrovascular amyloid deposits, Aβ is by far the most common [66], with specific mutations at the 21–23 hot spot in its sequence showing strong vascular compromise and association with early-onset CAA and lethal cerebral hemorrhages [1]. The presence of fibrillar amyloid deposits narrows the vessel lumen and changes its architecture, impairing cerebral blood flow and generating local hypoxic/ischemic conditions that negatively impact the metabolic regulation and function of the neurovascular unit, a structural and functional interactive association between neural cells and the cerebrovasculature that regulates cerebral blood flow and maintains the integrity of the BBB [67,68]. The progressive build-up of CAA triggers a secondary cascade of metabolic events, exacerbating oxidative stress damage, altering blood–brain barrier permeability, and increasing the risk of microhemorrhages, compromising the neurovascular unit in a vicious cycle.

Aβ fibrillar deposits are highly heterogeneous, composed of a mixture of peptides of different lengths and exhibiting numerous post-translational modifications that contribute to the poor solubility and perpetuation of the lesions [69,70,71]. As important as the detrimental effects on the cerebrovasculature exerted by fibrillar amyloid deposits, prefibrillar aggregates in the form of soluble Aβ oligomers are considered today the key elements associated with cytotoxicity, synaptic dysfunction, neuroinflammation, and cognitive impairment characteristic of AD. As such, these oligomeric assemblies have emerged as promising candidate biomarkers for early diagnosis and potential targets for novel therapeutic modalities [72,73,74,75,76]. In cerebral vessels, Aβ oligomers have been credited for the induction of endothelial and smooth muscle cells’ toxicity, mitochondrial dysfunction, generation of oxidative stress, and release of inflammatory mediators, all elements associated with disruption of the BBB permeability, impaired brain Aβ clearance, and formation of cerebral microbleeds [14,22,77,78]. It is clear that amyloidogenic proteins and peptides can adopt multiple conformations and numerous states of aggregation before they reach the stable Congo red- and Thioflavin S-positive amyloid fibrillar structures that form the classic parenchymal and vascular deposits. In the case of Aβ, numerous soluble oligomeric intermediate states have been described—micelles, protofibrils, amorphous aggregates, prefibrillar aggregates, and Aβ-derived diffusible ligands (ADDLs) [79,80,81,82,83,84]. Despite exhibiting variable molecular mass—ranging from dimeric structures to 60 kDa—these assemblies share common conformational epitopes that are recognized by the polyclonal antibody A11 used in our studies, which reacts with prefibrillar oligomers but is blind to monomers and mature fibrils [50]. Conversely, an antibody raised against fibrillar structures—polyclonal OC—also employed herein recognizes oligomeric structures of molecular mass of up to 250 kDa that overlap in size but are immunologically distinct from those immunoreactive with the A11 antibody [49]. In our experimental paradigm, conformational oligomers of both types were demonstrated in the Aβ1-42 and AβQ22 preparations. In contrast, no significant levels of either of these structural assemblies were detected, within the timeframe of our experiments, in the Aβ1-40 homologues, as indicated by the almost negligible immunoreactivity with both A11 and OC antibodies (Figure 1). These data indicate that, consistent with the CD and Thioflavin T binding assay results, Aβ1-42 and AβQ22 form soluble prefibrillar and fibrillar oligomers faster than Aβ1-40, suggesting the requirement of longer incubation times for Aβ1-40 oligomerization. In this sense, our previous electron microscopy studies showed that peptide fibrillization is time dependent, with Aβ1-40 generating protofibrils after six days of incubation in identical experimental conditions in which AβQ22 only required one day of incubation [43]. Further studies using pre-formed highly purified Aβ1-40 oligomers will be necessary to determine whether this peptide is also capable of assembling into the same type of fibrillar and prefibrillar oligomers shown herein for Aβ1-42 and AβQ22, as well as whether these oligomeric species or a different type of structures has the ability to induce ROS formation on microvascular EC.

The structural assemblies of Aβ1-42 and AβQ22 illustrated in Figure 1, in turn, were able to generate ROS formation in ECs after a 24 h exposure, as illustrated in Figure 2, whereas Aβ1-40 had no immediate effect in this timeframe, likely requiring a longer time for the generation of the harmful oligomeric species. Studies from our group and others have previously demonstrated the induction of ROS formation in neuronal cells upon challenge with Aβ1-42 in a comparable timeframe, suggesting the capability of Aβ peptides to elicit common paths in different cell types [35,42]. Despite not being a quantitative evaluation, ROS formation assessment by IF microscopy appears to indicate a stronger response in neurons [42] than in our experimental EC model, as suggested by the brightness of the images. The definite assessment of differential cell sensitivities to the Aβ peptides, changes in cell morphology, or in the proteins involved in specific cellular functions and downstream mechanisms would require conducting simultaneous experiments challenging the different cell types with comparable types of oligomeric assemblies. It is also important to emphasize that in our experimental paradigm, Aβ1-42 showed a comparable effect for ROS generation in ECs to the Dutch mutant AβQ22 primarily associated with hereditary CAA and cerebral hemorrhage. This strongly suggests that Aβ1-42, a central component of capillary CAA but a minor component associated with amyloid deposits in large vessels, has by itself the capability to initiate oxidative stress damage in ECs, with potential to alter BBB permeability, promote neuroinflammation, and increase the risk of developing microhemorrhages. This is an interesting issue that warrants further studies, particularly when the role of vascular amyloid in the pathogenesis of AD, neglected for many years, has started gaining attention.

Mitochondrial dysfunction plays a pivotal role in AD and has been described at early stages of the disease, as indicated by evidence of oxidative stress in mild cognitive impairment cases prior to the development of the neuropathological features characteristics of the disease [85,86,87,88]. As a consequence of its high oxygen consumption, amounting up to 20% of the total body levels, the brain is also the most important ROS generator and is particularly susceptible to oxidative stress damage due to its abundant lipid content and relatively low antioxidant capacity [89,90]. The fluorogenic probes employed in the current study do not target any specific ROS molecules, recognizing instead a variety of species, including the highly reactive hydroxyl radicals, which—as a consequence of their high reactivity—cannot act as substrates for any enzyme and are, therefore, primarily neutralized by interaction with adjacent oxidizable molecules, including amino acids and lipid molecules [91,92]. Lipids, as a result of the unsaturated bonds in the fatty acid chain, are especially vulnerable to oxidative stress, leading to the formation of lipid hydroperoxides, including MDA, 4-hydroxynonenal, and acrolein [44,93]. These lipid modifications, able to cause membrane lipid degradation and irreversible cell damage, are markers of oxidative stress reported in plasma and in association with neuropathological lesions in AD brains [44,92]. Our IF microscopy findings, using a fluorometric lipid sensor (Figure 3B,C), showed significant levels of lipid peroxidation in ECs challenged with Aβ1-42 and AβQ22 peptides, correlating with the elevated levels of MDA, a reactive aldehyde that results from the peroxidation of polyunsaturated fatty acids (Figure 3A). No significant changes in MDA levels were detected in control groups or in cells challenged with Aβ1-40, correlating with ROS formation illustrated by the IF and ELISA detection of ROS/RNS levels in WCLs (Figure 2), overall parallelling the structural characteristics of the synthetic homologues depicted in Figure 1. Detailed studies challenging EC with pre-formed Aβ1-40 oligomeric assemblies, as indicated above, will determine whether these structures are able to generate and maintain chronic steady-state levels of ROS capable of inducing oxidative stress downstream mechanisms and causing EC dysfunction, a very important unresolved issue taking into consideration the prevalence of Aβ1-40 in cerebrovascular deposits of sporadic AD and CAA.

Biochemically, protein carbonylation is an oxidative change that results in the formation of reactive aldehyde or ketone residues on proteins, impairing protein structure and function and leading to partial or total inactivation [94,95]. Increased protein carbonylation was reported in the hippocampus and inferior parietal lobule of AD brains correlating with established neuropathological features of the disease, although the studies lacked information regarding the association of this post-translational modification with vascular deposits [96,97,98,99]. Our work, measuring protein carbonylation via dot blot analysis and ELISA (Figure 4), demonstrated the formation of this chemical modification in Aβ-challenged EC. Notably—in difference to lipid peroxidation—the modification was not only induced in cells incubated with the highly prone to oligomerization Aβ1-42 and AβQ22 proteoforms, but also with Aβ1-40, suggesting either a higher sensitivity of the detection methodology or the requirement of a lower ROS threshold to induce protein carbonylation. Alternatively, a lower degradation rate of carbonyl-modified proteins compared to peroxidized lipids may contribute to the higher accumulation of carbonylated proteins and their increased detection rate [64,65]. Future work will identify the specific proteins undergoing carbonylation following Aβ challenge, individualize other ROS-mediated changes, including protein nitrosylation, and assess nucleic acid oxidative modifications, which have the potential to translate in DNA damage and genomic instability [100,101].

Redox imbalance is a crucial contributor to many pathological conditions, including AD. Under normal physiological conditions, the formation and elimination of free radicals is highly controlled by several endogenous systems developed to avoid oxidative stress and toxicity. These antioxidant mechanisms involve ROS scavengers, antioxidant enzymes—including superoxide dismutase, catalase, and glutathione peroxidase—and endogenous non-enzymatic antioxidants, such as glutathione [102]. A crucial regulator of the cellular responses protecting against oxidative and electrophilic stress is the transcription factor Nrf2. Under conditions of exacerbated ROS production and upon activation by different pathways, Nrf2 is translocated to the nuclei in which it binds to the antioxidant response elements (AREs) motifs to activate the transcription of numerous antioxidant genes [40,41,103,104,105]

Numerous compounds targeting diverse pathways of the antioxidant response have been tested in different clinical and experimental settings [41,93,106,107]. The field is quite complex, since in some instances, the mechanisms underlying the protective action of the agents are not completely understood, while in other cases some compounds exhibit a multifaceted action exerting their protective effects by interlinking different mechanisms. The α-tocopherol analog TX and the carbonic anhydrase inhibitor MTZ employed in the current work are agents with known antioxidant properties, acting through various mechanisms and that recently have also been reported as activators of Nrf2 [30,42,108,109]. Along this line, TX is known for its high radical scavenging activity of peroxyl and alkoxyl radicals and, as such, not only can it act directly by quenching ROS formation but it is also likely to activate Nrf2 by interfering with its binding to KEAP 1 [110,111]. MTZ is more well known for its action as a carbonic anhydrase inhibitor, but it is also a multitarget compound exhibiting broad capabilities. In addition to activating Nrf2 through inhibition of the PI3K/AKT axis, the compound exhibits antioxidant properties by protecting cells from H_2_O_2_-induced damage [38], inhibiting ROS formation, and restoring mitochondrial membrane potential [35,109]. Previous work from our group showed that both small molecules, TX and MTZ, increased the activation and nuclear expression of Nrf2, protecting neuronal cells from oxidative, metabolic, and bioenergetic changes [42]. The agents restored mitochondrial dysfunction induced by Aβ1-42 oligomeric forms, counterbalancing respiration alterations and ROS production while increasing the downstream expression of the antioxidant response proteins SOD-1 and HO-1 [42]. The current study expanded on previous findings, demonstrating that TX and MTZ efficiently inhibited Aβ-mediated ROS generation in microvascular ECs [42,110,112]. The decrease in Aβ-mediated production of free radicals in ECs observed after treatment with TX and MTZ also led to a concomitant reduction in oxidative stress damage, as evidenced by decreased levels of lipid peroxidation and a reduction in the formation of protein carbonyl derivatives. Despite the indisputable role of TX and MTZ in ameliorating ROS formation and the concomitant oxidative stress demonstrated herein, the extent to which any of their reported multitarget functions exerts a more prominent role in their protective action remains to be definitively determined. Certainly, the future use of Nrf2-silencing mRNA methodologies would be a useful tool in providing an irrefutable demonstration of the contribution of the transcription factor to the protective antioxidant properties of TX and MTZ.

## 5. Conclusions

**Figure 5:**
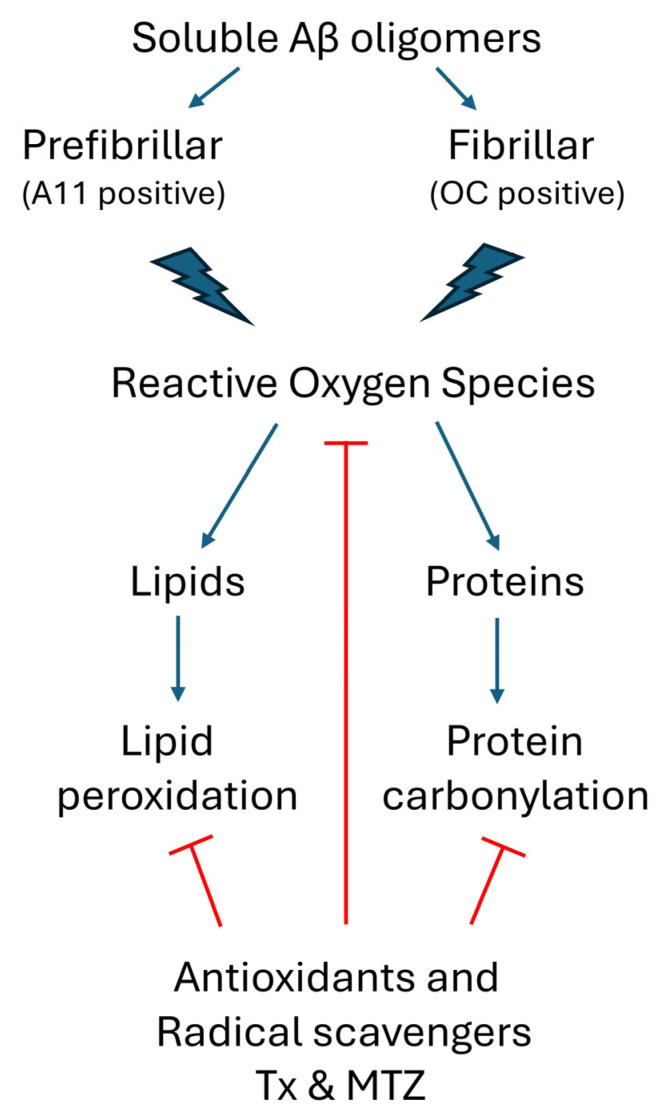
Schematic representation of soluble oligomeric Aβ-mediated ROS generation and the antioxidant cellular response. Prefibrillar A11-positive Aβ oligomers and fibrillar Aβ OC-positive oligomers induce the production of ROS in cerebral microvascular endothelial cells. In turn, these free radical species target cellular lipids and proteins, generating undesirable lipid peroxides and protein carbonyls, both markers of oxidative stress. Pretreatment of the cells with antioxidants TX and MTZ quenches ROS formation, reducing the generation of these chemical modifications to background levels. (Red lines indicate points of inhibition for the antioxidants Txand MTZ).

The detrimental contribution of amyloid deposition in cerebral vessels and its morphological and functional vascular changes is increasingly recognized as an important contributor to AD pathogenesis. The mechanisms leading to the formation of amyloid deposits as well as the detailed pathways ultimately responsible for the endpoint cellular dysfunction are highly complex and interlink different molecular pathways. The propensity of the amyloidogenic Aβ peptide to form fibrillar deposits is exacerbated by the presence of mutations affecting the proclivity to generate β-structures favoring peptide aggregation, toxicity, and alteration of mitochondrial function and ROS homeostasis, contributing to the disease pathogenesis in multiple ways. Adding to this complexity is the active participation of conformationally distinct Aβ oligomeric structures as triggers of pathogenic molecular events, as highlighted in the current work. Understanding which of these antigenically different but somehow interconnected structures is responsible for the pathogenic process in cerebral endothelial cells and whether the same conformational requirements could be extended to other brain cell populations remain to be determined. Overall, as illustrated in Figure 5, the work presented here provides insight into the effects induced by a combination of soluble fibrillar and prefibrillar Aβ oligomers associated with sporadic and familial forms of AD on microvascular ECs, resulting in ROS production and consequent oxidative effects, as shown by the presence of lipid peroxides and protein carbonyls. Pretreatment with antioxidants significantly attenuated ROS generation and concomitant ROS-induced damage, validating the relevance of their potential use for pharmacological intervention. Based on the complexity of the molecular mechanisms involved in AD, it is increasingly evident that future successful therapeutic modalities will likely require complex strategies targeting multiple pathways. The use of antioxidants, radical scavengers, and/or small molecule Nrf2 activators may provide additional/alternative approaches in combinatorial therapies with the potential to mitigate the Aβ-mediated mitochondrial dysfunction and preserve the integrity of the neurovascular unit.

## Data Availability

The original contributions presented in this study are included in the article and Supplementary Material. Further inquiries can be directed to the corresponding authors.

## Supplementary Materials

The following supporting information can be downloaded at: https://www.mdpi.com/article/10.3390/antiox14111375/s1, Figure S1: Microvascular endothelial cells challenged with sporadic andfamiliar Aβ species. Representative images of Human brain microvascular endothelial cells (hCMEC/D3) challenged for 24 h or 48 h with either Aβ1-42, Aβ1-40 or AβQ22 peptides at a finalconcentration of 50 µM, Phase contrast images were captured in a Nikon Eclipse Ti invertedmicroscope. In all cases bars represent 100 µm.
