# Supplementary material for: Antioxidants Trolox and Methazolamide Protect Microvascular Endothelial Cells from Oxidative Damage Induced by Sporadic and Familial Forms of Oligomeric Amyloid-β
Source: Antioxidants (Basel). 2025 Nov 19;14(11):1375. doi: 10.3390/antiox14111375 (PMC12649480; doi:10.3390/antiox14111375)
Supplement: Supplementary file 1 [file antioxidants-14-01375-s001.zip › antioxidants-3831747-supplementary.pdf]

Supplementary

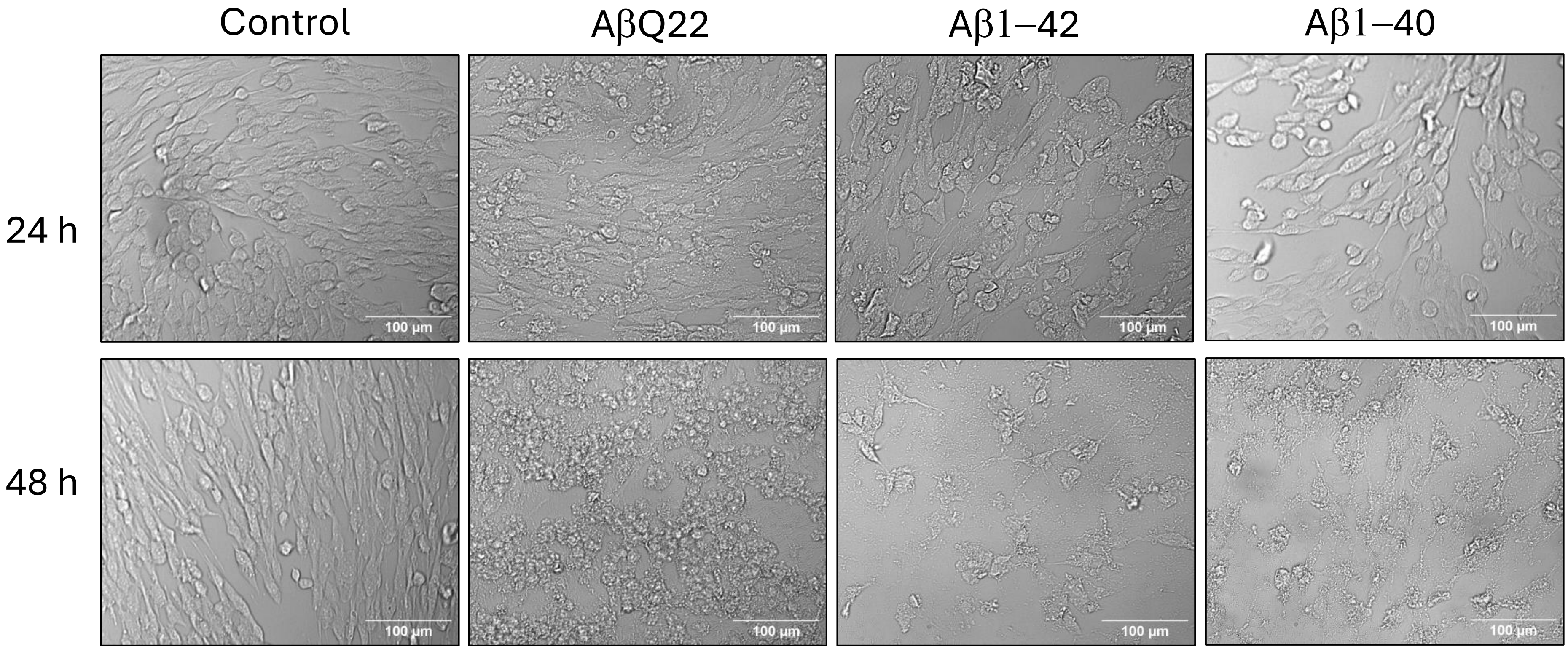

Supplementary Figure S 1. Microvascular endothelial cells challenged with sporadic and familiar Aβ species. Representative images of Human brain microvascular endothelial cells (hCMEC/D3) challenged for 24 h or 48 h with either Aβ1-42, Aβ1-40 or AβQ22 peptides at a final concentration of 50 μM. Phase contrast images were captured in a Nikon Eclipse Ti inverted microscope. In all cases bars represent 100 μm.
